# Characterization and Summarization of the Impact of Electronic Cigarettes on the Cardiovascular System: A Systematic Review and Meta-Analysis

**DOI:** 10.7759/cureus.39528

**Published:** 2023-05-26

**Authors:** Ali Rahman, Sura Alqaisi, Rana Alzakhari, Sunil Saith

**Affiliations:** 1 Internal Medicine, Northwell Health at Mather Hospital, Port Jefferson, USA; 2 Internal Medicine, Memorial Healthcare, Pembroke Pines, USA; 3 Internal Medicine, Richmond University Medical Center, Staten Island, USA; 4 Cardiology, State University of New York Downstate Medical Center, Brooklyn, USA

**Keywords:** heart failure, qtc interval, myocardial infarction, cardiovascular diseases, e-cigarettes, electronic cigarettes

## Abstract

Electronic cigarettes may increase the risk of long-term cardiovascular morbidity. To protect the heart, awareness should be raised of the risks and limits of E-cigarette aerosol exposure. Thus, this systematic review and meta-analysis assessed the cardiovascular risk of e-smoking. This systematic review was conducted by using the Preferred Reporting Items for Systematic Review and Meta-Analysis (PRISMA) statement. We searched PubMed, Embase, Scopus, Web of Science, and Science Direct databases in December 2022 to identify studies investigating e-cigarettes' impact on the heart. The study was supported by meta-analysis and qualitative review.

Out of the initial 493 papers, only 15 met the inclusion criteria and were included in the study. The cumulative number of participants in the myocardial infarction (MI) group was 85,420, and in the sympathetic groups in whom the systolic blood pressure (SBP), diastolic blood pressure (DBP), mean blood pressure (MBP), and heart rate (HR) were measured, were 332 cigarette smokers. The control group included the “never use,” “non-smokers,” and “never smoke.” The pooled analysis showed a significant difference between the e-cigarette smokers and the control group regarding the risk of developing MI in former smokers (OR= 0.12; 95% CI: 0.01-1.72, P = 0.12) and never smoked (OR= 0.02; 95% CI: 0.00-0.44, P = 0.01) favoring the control group. The pooled analysis of the included studies showed a significant difference between the e-cigarette smokers with nicotine and the control group regarding the mean difference (MD) of the SBP (MD = 2.89; 95% CI: 1.94-3.84; P < 0.001), the DBP (MD = 3.10; 95% CI: 0.42-5.78; P = 0.02), the MBP (MD = 7.05; 95% CI: 2.70-1.40; P = 0.001), and HF (MD = 3.13; 95% CI: 0.96-5.29; P = 0.005) favoring the control group. We conclude that using e-cigarettes has a detrimental effect on cardiac health. The risk of severe cardiac conditions increases with e-cigarettes. Thus, vaping can do more harm than good. Consequently, the misleading notion that e-cigarettes are less harmful should be challenged.

## Introduction and background

The Centers for Disease Control and Prevention have reported that electronic cigarettes are associated with an increased risk of cardiovascular disease [1]. E-cigarette vapor includes particles that may enter the circulation and promote inflammation. These particles increase the risk of stroke, myocardial infarction (MI), and other cardiovascular events [2-3]. E-cigarettes also contain nicotine which is the main psychoactive component of e-cigarettes and is known to be a vasoconstrictor that can increase blood pressure and heart rate [4-5]. This can stress the cardiovascular system and increase the risk of heart attack and stroke [6]. In addition, some of the chemicals in e-cigarette aerosol can damage the endothelium [7], leading to a buildup of atherosclerotic plaque, which can block or reduce blood flow [8]. This can cause serious cardiovascular problems, including arrhythmias [9], stroke [10], and MI [11]. To sum up, e-cigarettes pose a significant risk to cardiovascular health, leading to long-term complications.

Nicotine is considered the main culprit in cigarette-attributed cardiovascular devastation. The hemodynamic impact of nicotine is mediated by the activation of the sympathetic nervous system with the consequent release of noradrenaline and adrenaline in excess [12]. As a result of adrenaline release, cardiac work is augmented by the stimulation of heart rate, myocardial contractility, and blood pressure [13-14]. In patients with coronary insufficiency, nicotine impairs coronary blood supply and induces coronary spasms, thus increasing the risk of myocardial ischemia [15]. Moreover, nicotine has been associated with heart failure by promoting myocardial remodeling leading to hypertrophy and fibrosis [15].

Furthermore, the nicotine-mediated catecholamine releases may contribute to fatal cardiac arrhythmias [16]. In addition, some studies suggested that nicotine can increase platelet activation and hence, the risk of arterial thrombosis [17]. There is evidence that nicotine impacts endothelial function by enhancing oxidative stress and chronic inflammation [18].

Nicotine replacement therapy (NRT) [19] provides the body with a small, controlled amount of nicotine, which helps reduce cravings and other withdrawal symptoms that occur when quitting smoking [20]. NRTs come in patches, gum, sprays, inhalers, and lozenges, and the amount of nicotine in each form varies. NRTs are designed to reduce the severity of nicotine withdrawal symptoms, allowing the user to focus on the psychological aspects of quitting smoking. NRT has been shown to double the chances of successful quitting [21] nearly. To improve quitting success rates, NRT [22] may be used with other methods, including counseling and behavioral therapy [23]. It is well-accepted that NRT has no significant health risks [24]. On the other hand, NRT seems to have favorable links with MI, mortality, and suicidal ideation [20].

There is an ongoing controversy concerning the possible hazards and advantages of e-cigarette usage. E-cigarette advocates claim that the devices may be a safer alternative to regular cigarettes and might assist smokers in quitting [25]. However, evidence shows that e-cigarette devices are hazardous and may raise the risk of cardiovascular disease [4]. Furthermore, there are worries that e-cigarettes may be a gateway for young people to conventional cigarette usage and nicotine addiction [26]. In addition, there is evidence that using e-cigarettes might lead to inhaling potentially toxic substances, such as formaldehyde and metals [27]. The possible hazards and advantages of e-cigarette usage are the subject of continuing controversy. While some claim that the devices may be a safer alternative to regular cigarettes and might assist smokers in quitting, others contend that the devices are harmful and can raise the risk of cardiovascular disease.

## Review

Methods

The Preferred Reporting Items for Systematic Reviews and Meta-Analyses (PRISMA) statement guidelines [19] were followed throughout the processing stages of this study. The processing stages were performed according to the Cochrane Handbook for Systematic Reviews of Interventions [22-28]. 

Eligibility criteria 

The criteria for included studies were the following: Studies with age groups more than 18, including randomized controlled trials (RCTs), cross-sectional studies, and clinical trials; studies concerning the impact of e-cigarettes on the cardiovascular system; studies published in English; studies on humans.

The exclusion criteria encompassed the following studies: Studies on children under 18 years of age; studies on animals; non-clinical trial articles such as reviews, clinical conferences, editorials, clinical study protocols, case studies; studies concerning systems other than the cardiovascular system.

Outcomes 

The primary quantitative outcomes include sympathetic system activation and MI. The secondary qualitative outcomes include heart failure and cardiac electrical conductivity presented by QTc.

Endpoints 

The systematic review includes both quantitative and qualitative analysis of data. 

Search strategy and study selection 

PubMed, Scopus, Web of Science, Embase, and Science Direct databases were searched using relevant keywords in January 2023. The search terms include electronic cigarettes, e-cigs, e-cigarettes, ECIG, vapes, the cardiovascular system, heart failure, MI, QTc, electrocardiogram (ECG), endothelial, and sympathetic. Wildcards and operators (AND, OR, NOT) intensified the search. MeSH term searches were used when applicable.

The search results were screened, and publications were selected depending on the selection criteria. The exclusion of the publications was based first on the paper's title, then the abstract, and finally, the retrieved full text. The publications' bibliographic references were manually examined to identify any other acceptable studies that may have been overlooked in earlier phases. For further processing, data were extracted and compiled in an electronic spreadsheet. The retrieved data comprised demographic information, age groups, gender, smoking pattern (ex-smoker, e-cigarette smoker, and dual smoker), smoking mode (tobacco smoking or e-cigarette smoking), and the present morbid status vs. healthy individuals.

Quality assessment 

For appraising the evidence, the quality assessment tool proposed by Hawker et al. (2002) was utilized in the current study to determine the quality of the included studies [29]. The protocol design by Hawker et al. (2002) is composed of nine items, including abstract and title, introduction and aim, methods and data, sampling, data analysis, ethics and bias, results, transferability (generalizability), and implications (usefulness). Each item is valued as "good," "fair," "poor," and "very poor." The total and subtotal scores were presented to give a clear idea of the strength and weaknesses of each article based on the proposed criteria. 

Data extraction 

Data was obtained from texts, tables, and figures using Graph Grabber, version 2.0 (Quintessa, Henley-on-Thames, UK) as well as supplementary data. We focused on the outcome measures that included sympathetic system activity, including the heart rate and blood pressure, the risk of MI and heart failure, and the QTc abnormality.

Statistical analysis 

This meta-analysis used the computer program Cochrane RevMan, version 5.4 (Cochrane, London, UK). The study groups include e-cigarette smoking, tobacco smoking, dual cigarette smoking, and former smokers, as well as e-cigarettes with and without nicotine. The "never use," "non-smokers," and "never smoke" are designated as the control group.

Regarding the study outcomes, the odds ratio (OR) with a 95% confidence interval (CI) was used for dichotomous variables where appropriate, while the mean difference (MD) and 95% CI were presented for continuous variables. Cochrane's P values and I2 were tested to examine heterogeneity among the studies [22]. The random effects model was adopted in this meta-analysis even if I2 was small to meet the expected high heterogeneity. Funnel plots and the Egger regression test could not be performed due to the limited number of included trials [30]. In addition, a sensitivity analysis was performed by sequentially deleting trials to check the stability of the outcomes. No significant effect happened after completing the sensitivity analysis due to the high variation between the included studies.

Results

*Literature Search Results* 

**Figure 1:**
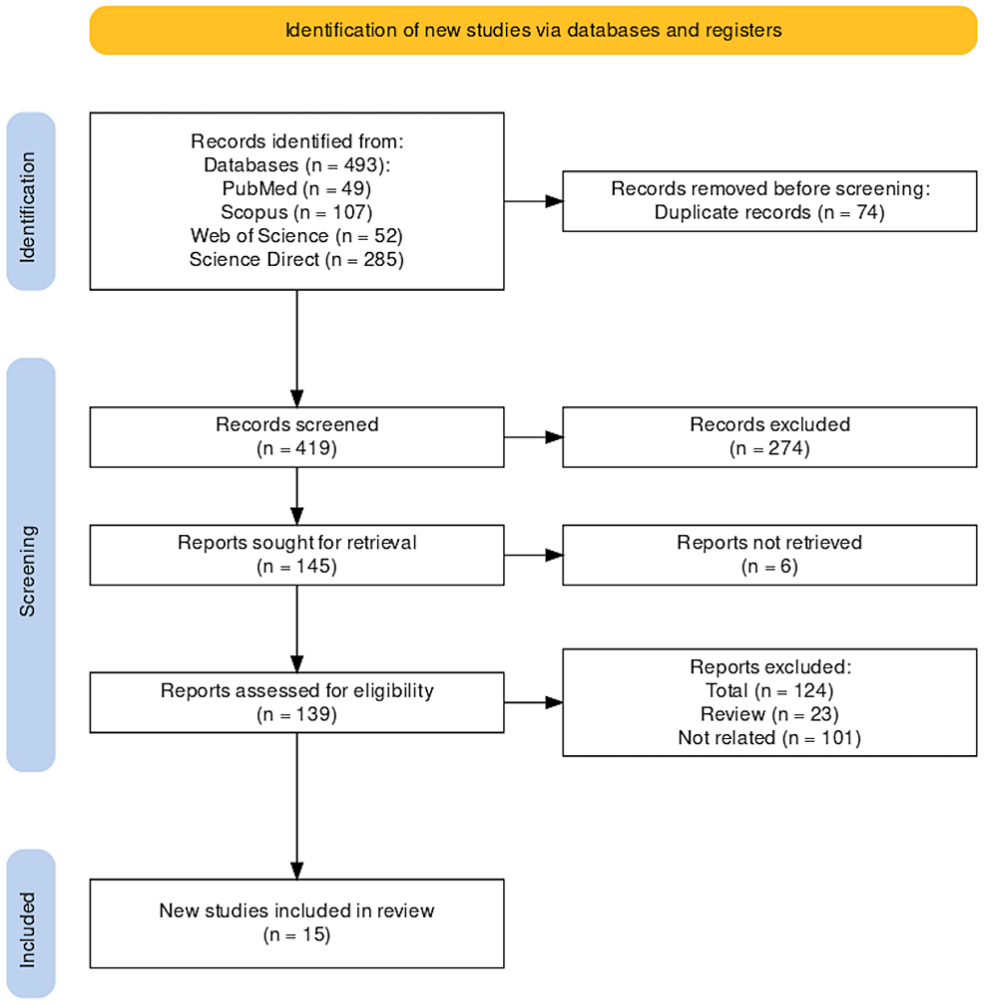
The Preferred Reporting Items for Systematic Reviews and Meta-Analyses (PRISMA) flow diagram.

The initial search yielded 493 papers from four databases, including PubMed, Scopus, Web of Science, and Science Direct. Of them, 74 articles were excluded due to duplication. Subsequently, 419 papers were subject to title and abstract screening, and 274 papers were excluded because they did not meet the inclusion criteria. The remaining 145 papers underwent full-text screening. A total of 15 studies were finally included for the final quantitative and qualitative analysis: eleven papers on the sympathetic activity of the heart [7-9], [31-39], three papers on MI in individuals using e-cigarettes [39-41], one paper on heart failure in individuals using e-cigarettes [42], and two papers on QTc interval changes in individuals using e-cigarettes [9-43] (Figure 1).

Descriptive Data 

The cumulative number of participants in the MI group was 85420 cigarettes smoker subdivided into four groups: current e-cigarettes smokers (n= 2663), current tobacco cigarettes smokers (n= 28481), dual cigarettes smokers (n= 8690), and former tobacco cigarettes smokers (n= 45586). The cumulative number of participants in the "sympathetic" group was 332 cigarettes smoker subdivided into four groups: current e-cigarettes smokers with nicotine (n=64), current e-cigarettes smokers without nicotine (n=64), and current e-cigarette smokers (irrespective of the nicotine contents) (n= 99), and current tobacco cigarettes smokers (n=105). The other two groups were subjected to qualitative review, including the heart failure and QTc interval groups.

Outcomes

Myocardial Infarction in Individuals Using E-cigarette Smoking

**Figure 2:**
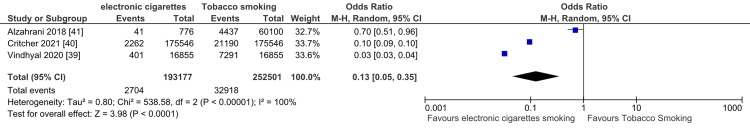
Forest plot: odds ratio of myocardial infarction in electronic cigarette vs. tobacco smoking.

The pooled analysis of the included studies showed a significant difference between the e-cigarette and tobacco smokers regarding the risk of developing MI (OR= 0.13; 95% CI: 0.05-0.35, P < 0.001). The pooled studies were heterogeneous (I2 = 100%; P < 0.001), and the heterogeneity could not be resolved by leaving one out (Figure 2).

**Figure 3:**
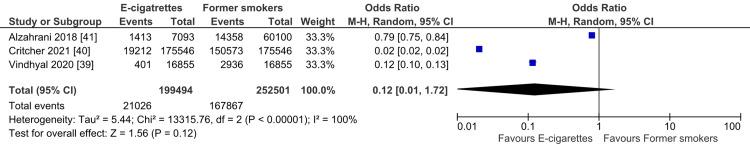
Forest plot: myocardial infarction in electronic cigarette smokers vs. former smokers.

The pooled analysis of the included studies showed a non-significant difference between the e-cigarette smokers and the former smokers regarding the risk of developing MI (OR= 0.12; 95% CI: 0.01-1.72, P = 0.12). The pooled studies were heterogeneous (I2 = 100%; P < 0.001), and the heterogeneity could not be resolved by leaving one out (Figure 3).

**Figure 4:**
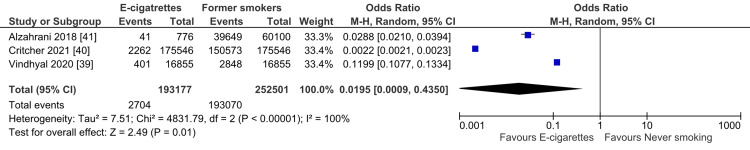
Forest plot: myocardial infarction in electronic cigarettes smokers vs. never smoking.

The pooled analysis of the included studies showed a significant difference between the e-cigarette smokers and the never smoking regarding the risk of developing MI (OR= 0.02; 95% CI: 0.00-0.44, P = 0.01). The pooled studies were heterogeneous (I2 = 100%; P < 0.001), and the heterogeneity could not be resolved by leaving one out (Figure 4).

Sympathetic activity with e-cigarette smoking

Systolic Blood Pressure Changes in Individuals Using E-cigarettes

**Figure 5:**
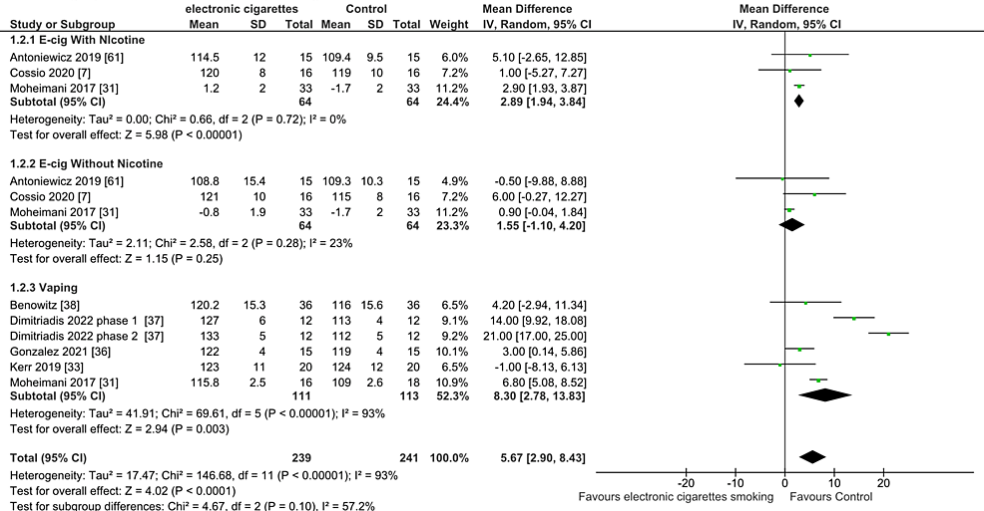
Forest plot: mean difference in systolic blood pressure with electronic cigarette smoking.

The pooled analysis of the included studies showed a significant difference between the e-cigarette smokers with nicotine and the control group regarding the mean difference (MD) of the systolic blood pressure (MD = 2.89; 95% CI: 1.94-3.84; P < 0.001) favoring the control group. The pooled studies were not heterogeneous (I2 = 0%; P = 0.42). As a result, electronic cigarettes had a more substantial negative influence on systolic blood pressure than the control group (Figure 5).

The pooled analysis of the included studies showed no significant difference between the e-cigarette smokers without nicotine and the control group regarding the MD of the systolic blood pressure (MD = 1.55; 95% CI: -1.10-4.20; P = 0.25). The pooled studies had low heterogeneity (I2 = 23%; P = 0.28). Thus, the effects of nicotine-free vaping on SBP were compared to those of a placebo group 

The pooled analysis of the included studies showed a significant difference between the e-cigarette smokers (vaping irrespective of the nicotine content) and the control group regarding the MD of the systolic blood pressure (MD = 8.30; 95% CI: 2.78-13.83; P = 0.003). The pooled studies were highly heterogeneous (I2 = 93%; P < 0.0001). There was a high degree of heterogeneity across the studies, even though vaping was more negatively impactful on SBP than control, regardless of whether or not nicotine was present 

The pooled analysis of three groups showed a significant mean difference in the systolic blood pressure with smoking e-cigarettes with and without nicotine and e-cigarette use compared to control groups (MD = 5.67; 95% CI: 2.90-8.43; P < 0.001). The pooled studies were heterogeneous (I2 = 93%; P < 0.0001), and the heterogeneity could not be resolved by leaving one out. The subgroup differences showed moderate heterogeneity (I2 = 57.2%; P= 0.10).

Diastolic Blood Pressure Changes in Individuals Using E-Cigarettes

**Figure 6:**
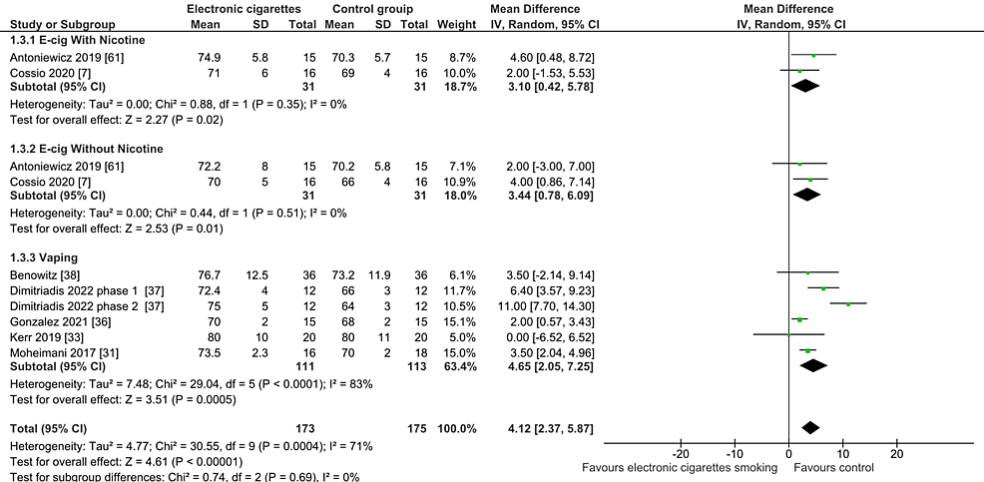
Forest plot: mean difference in diastolic blood pressure with electronic cigarette smoking.

The pooled analysis of the included studies showed a significant difference between the e-cigarette smokers with nicotine and the control group regarding the mean difference (MD) of the diastolic blood pressure (MD = 3.10; 95% CI: 0.42-5.78; P = 0.02) favoring the control group. The pooled studies were not heterogeneous (I2 = 0%; P = 0.35). As a result, electronic cigarettes had a more substantial negative influence on diastolic blood pressure than the control group (Figure 6).

The pooled analysis of the included studies showed a significant difference between the e-cigarette smokers without nicotine and the control group regarding the MD of the diastolic blood pressure (MD = 3.44; 95% CI: 0.78-6.09; P = 0.01) favoring the control group. The pooled studies were not heterogeneous (I2 = 0%; P = 0.51). Thus, vaping was negatively impactful on diastolic blood pressure even without the presence of nicotine.

The pooled analysis of the included studies showed a significant difference between the e-cigarette smokers (irrespective of the nicotine content) and the control group regarding the MD of the diastolic blood pressure (MD = 4.65; 95% CI: 2.05-7.5; P < 0.0001) favoring the control group. The pooled studies were moderately heterogeneous (I2 = 83%; P < 0.001). Therefore, vaping negatively affected diastolic blood pressure more than the control.

The overall pooled analysis of 4 groups showed a significant MD of the diastolic blood pressure with smoking e-cigarettes with and without nicotine and tobacco and e-cigarette use compared to control groups (MD = 4.12; 95% CI: 2.37-5.87; P < 0.0001). The pooled studies had low heterogeneity (I2 = 71%; P < 0.001). The subgroup differences showed no heterogeneity (I2 = 0%; P= 0.69).

Mean Blood Pressure Changes in Individuals Using E-Cigarettes

**Figure 7:**
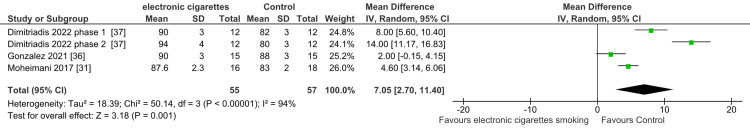
The mean difference of the mean blood pressure with electronic cigarette smoking.

The pooled analysis of the included studies showed a significant difference between the e-cigarette smokers and the control group regarding the MD of the mean blood pressure (MD = 7.05; 95% CI: 2.70-1.40; P = 0.001) favoring the control group. The pooled studies were of high heterogeneity (I2 = 94%; P < 0.001) (Figure 7). Accordingly, vaping negatively impacted the MBP more than the control group.

Heart Rate Changes in Individuals Using E-Cigarettes

**Figure 8:**
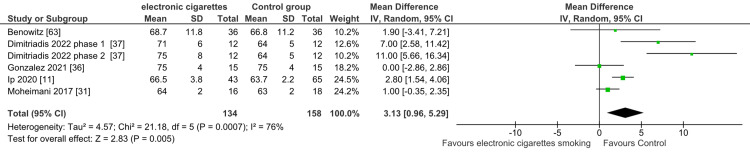
Forest plot: mean difference in the heart rate with electronic cigarette smoking.

The pooled analysis of the included studies showed a significant difference between the e-cigarette smokers and the control group regarding the MD of heart failure (MD = 3.13; 95% CI: 0.96-5.29; P = 0.005), favoring the control group. The pooled studies had moderate heterogeneity (I2 = 76%; P = 0.0007). Consequently, vaping was found to have a negative impact on HR compared to the control (Figure 8).

Qualitative data analysis

Heart Failure in Individuals Using E-cigarettes

In an American study of 484 participants (adults with heart failure), e-cigarettes were used only by 1% of the participants. Only 5% of heart failure participants were dual users because they believed e-cigarettes cause less harm. That is to say e-cigarettes are not safe. Therefore, those at risk of serious outcomes avoid using e-cigarettes. It was recommended to assess and monitor the use of e-cigarettes as a harm-reduction approach [42].

QTc Interval in Individuals Using E-cigarettes

In the United States (US), heart rate variability and corrected QT interval measures were used to screen for secondhand smoke among five otherwise healthy volunteers with no cardiovascular illness history. In a randomized, repeated measures crossover study of healthy nonsmoking volunteers, it was concluded that short-term exposure to secondhand e-cigarettes emissions was associated with a significant reduction in the mean of the standard deviation of normal-to-normal intervals (SDNN), the average of the SDNN (ASDNN) intervals, the root mean square of successive differences (rMSSD), QTc interval, and heart rate. The reduction of the heart rate variability measures and the QTc interval was associated with the nicotine content of the e-cigarettes [43].

ECG indices of ventricular repolarization (Tpeak to Tend(Tp-e), Tp-e/QT, and Tp-e/QTc) were significantly longer in tobacco smokers than e-cigarettes smokers with nicotine, according to the findings of a study carried out in the US on 145 adult participants. These participants included long-term e-cigarette and tobacco product users and individuals who had never smoked tobacco. The adverse impact of tobacco and e-cigarettes on ventricular repolarization was associated with a rise in the risk of sudden death [9].

Discussion

The long-term health consequences of consuming electronic cigarettes are yet unclear. Evidence, however, suggests they may be detrimental to the cardiovascular system [44]. E-cigarettes are meant to appear and feel like conventional cigarettes and are often used by those attempting to quit smoking [45]. Studies have shown that e-cigarettes can increase the risk of MI and heart failure with an increased risk of mortality due to the nicotine content - a highly addictive substance [46]. When individuals start using e-cigarettes, they develop tolerance, requiring progressively higher nicotine levels to experience the same stimulation. This may eventually drive individuals to progressively increase their nicotine dose (by excessive smoking), resulting in addiction and reliance on e-cigarettes, which can raise the risk of serious cardiovascular events [13-47]. In addition, e-cigarettes contain other harmful chemicals, such as metals and chemicals that can damage the lungs [48]. Therefore, switching to e-cigarettes should be based on weighing the risks. Matching with the literature, the findings of our study showed that e-cigarette smoking confers less risk of MI compared to tobacco smoking. However, those who smoked e-cigarettes had a higher risk of MI than those who had never smoked or had quit smoking. This suggests that vaping may prevent MI compared to traditional tobacco smoking. However, there is still a risk of MI associated with e-cigarette use. On the other hand, the studies carried out were heterogeneous, reflecting the great differences in sampling and design.

The findings of the qualitative review revealed that continuous exposure to e-cigarette emissions with nicotine enhances sympathetic predominance, which was demonstrated by decreased heart rate variability measures and lengthening of the QTc interval, indicating a rise in cardiovascular risk [43]. In addition, it was concluded that e-cigarettes with nicotine, rather than without nicotine, prolong the ECG indexes of ventricular repolarization comparable to tobacco cigarette smoking [9]. Prolonging the ECG indexes of ventricular repolarization reflects the increased susceptibility to ventricular arrhythmias [49] with an increased risk of sudden death [50].

Despite the increasing evidence of increased HF risk with e-cigarette smoking [51], only one study was conducted in the last five years. In this study, e-cigarettes were used by patients with heart failure because of the expected reduced harm to nearby individuals and themselves [42]. It was postulated that the risk of heart failure is increased by increased risk factors, including arterial blood pressure, heart rate, and atherosclerosis [18-52].

The literature suggests that tobacco and e-cigarettes release nicotine to blood circulation, activating the sympathetic nervous system [46-53]. The nicotine-derived sympathetic activation is emphasized to impact the cardiovascular system causing an increase in blood pressure, heart rate, myocardial contractility, and coronary spasm [54].

In this meta-analysis study, the impact of e-cigarettes, especially nicotine, on the sympathetic activity of the heart was obvious regarding SBP, DBP, MBP, and HR. E-cigarettes significantly elevate systolic blood pressure irrespective of the nicotine content in the e-cigarettes subgroup. On the other hand, the impact of e-cigarettes on diastolic blood pressure was more robust. In addition, e-cigarette use was significantly associated with increased mean blood pressure. In accordance with the increase in sympathetic activity, e-cigarettes were found to increase HR significantly.

The findings of this study match the recent literature. A recent study showed that both heart rates and mean blood pressure increase during vaping, and the MBP do not decrease in recovery, negatively affecting the muscle sympathetic nerve activity [36]. Compared to tobacco cigarette use, e-cigarettes acutely elevate blood pressure and HR, an effect attributable to nicotine rather than non-nicotine use [55]. Therefore, the excitatory effect of e-cigarettes on the heart has been well established [56]. However, recent studies have only focused on contrasting traditional and electronic cigarettes to conclude that the latter has fewer health risks [57]. The current body of evidence on the impacts of electronic cigarettes on the cardiovascular system is of utmost concern [58]. Being less harmful than conventional tobacco cigarettes does not confer being harmless [54-59]. Unless supported by stronger evidence, the electronic cigarette should not be assigned as a cardiovascular-safe product [60-61].

Methodological quality assessment was done on all the included studies (Table 1) using the Hawker Appraisal Tool [29]. All the included studies had a high-quality assessment score of 78-100%. Of the total 15 studies assessed, nine attained a score of 100%, while only one study scored less than 80% with a minimum score of 28. Thus, the studies showed a minimal risk of bias in the overall evidence.

**Table 1:** Hawker appraisal of the selected papers.

Author/year	1- Abstract and title	2-Introduction and aims	3-Method and data	4-Sampling	5-Data analysis	6-Ethics and bias	7-Results	8-Transferability	9-Implications	scoring	percentage
Alzahrani T et al. (2018) [41]	4	4	4	4	4	4	4	4	4	36	100
Antoniewicz et al. (2019) [61]	3	4	4	3	3	4	3	3	3	30	83
Benowitz et al. (2020) [38]	4	4	4	4	4	4	4	4	4	36	100
Cossio et al. (2020) [7]	2	2	2	3	4	4	3	4	4	28	78
Critcher and Siegel (2021) [40]	4	4	4	4	4	4	4	4	4	36	100
Dimitriadis et al. (2022) [37]	3	4	4	4	4	4	4	4	4	35	97
Gathright et al. (2020) [42]	3	3	4	4	4	4	3	2	3	30	83
Gonzalez and Cooke (2021) [36]	4	4	4	4	4	4	4	4	4	36	100
Hiler et al. (2017) [35]	4	4	4	4	4	4	4	4	4	36	100
Hiler et al. (2020) [34]	4	4	4	4	4	4	4	4	4	36	100
Ip et al. (2020) [9]	4	4	4	4	4	4	4	4	4	36	100
Kerr et al. (2019) [33]	4	4	3	4	3	4	3	3	3	31	86
Lee et al. (2019) [43]	4	4	4	4	4	4	4	4	4	36	100
Moheimani et al. (2017) [31]	4	4	4	4	4	4	4	4	4	36	100
Vindhya et al. (2020) [39]	3	3	3	4	4	4	4	4	4	33	92

## Conclusions

The current systematic review and meta-analysis with qualitative review emphasize the detrimental effects of e-cigarettes on the cardiovascular system. The findings of this review concurred with the previous literature on raising awareness of the risks of e-cigarettes on the heart. E-cigarettes impose a cardio-excitatory effect on the heart with an increased risk of heart failure and coronary insufficiency. There is published evidence that e-cigarettes should not be considered safe and harmless. Therefore, e-cigarettes should not be encouraged in patients with compromised cardiovascular systems.
